# Moral character and competence judgments of sexual harassers and fraudsters in academic and business contexts

**DOI:** 10.1371/journal.pone.0312930

**Published:** 2024-11-12

**Authors:** Katarzyna Miazek, Konrad Bocian, Katarzyna Myslinska-Szarek

**Affiliations:** Department of Psychology in Sopot, SWPS University, Warsaw, Poland; University of Padova, ITALY

## Abstract

Fraud and sexual harassment have been haunting academia for years. While the scientific community proposed strategies to overcome misconduct in research, the problem of sexual harassment seems unresolved. One reason for this might be a difference between men and women in the perception of the moral character and competence of sexual harassers. Across four studies (*N* = 3776), in the UK and the US, men judged the sexual harasser as less immoral than women (Studies 1, 2, and 3a), even though sexual harassment was considered more harmful than fraud (Study 2). Consequently, men demanded less punishment for sexual harassers than women (Studies 1 and 2). This gender difference was not explained by moral rationalization (Study 3a). Further, a sexual harasser was judged as more competent than a fraudster but in an academic, not business, context (Studies 1 and 2). This effect was driven by the moral decoupling process, which participants used to separate competence judgments from moral judgments (Study 3b). Overall, these results suggest that in the academic context, gender interests most likely shape moral and punishment judgments towards sexual harassers, while the decoupling process allows both genders to perceive them as competent and immoral at the same time.

## Introduction

The #MeToo movement ignited worldwide discussions on sexual harassment, with academic institutions not being exempt. Nevertheless, universities face difficulties unmasking and removing harassers from institutions [1]. A review by Bondestam and Lundqvist [2] suggests that almost no evidence supports the efficacy of measures to prevent sexual harassment in higher education. At the same time, the scientific community proposed several strategies to mitigate scientific misconduct, such as the establishment of exemplary practices [3], research guidelines [4], and new journal submission formats [5], among other initiatives. While fighting fraud seemed to result in concrete steps toward greater scientific integrity, there are no unifying international policies or processes directly targeting sexual harassment. This paper attempts to understand this discrepancy by investigating how public opinion judges sexual and fraud transgressors in academic and business contexts.

Specifically, we aim to explore how people perceive the morality and competence of sexual harassers and fraudsters and whether context (business vs. academic) impacts these social perceptions. In other words, we aim to examine how people perceive transgressors as social beings (their morality, e.g., how trustworthy they are) and their capability to attain goals (their competence, e.g., how capable they are). As we explain further, both are crucial in how people perceive and interact with others around us. We focus on moral character perceptions because they impact trustworthiness and perceived suitability for social roles, which are critical in our daily interactions [6]. We also attempt to unpack the psychological process involved in the moral and competence perceptions to understand how these two traits impact global impressions of sexual offenders and fraudsters. Finally, since the recent increase in sexual harassment and fraud cases in the business world has led to heightened public awareness, we explore if there is a difference in how people perceive both offenders in academic and business contexts.

### The scope of the problem

The social science community was shocked after discovering that Diederick Stapel had fabricated research for years [7]. Unfortunately, Diderick’s case was not the only one. In psychology, 0.82 per 10,000 journal articles were retracted due to scientific misconduct [8]. Moreover, one in two academics admitted to frequently engaging in at least one questionable research practice, and one in twelve reported having falsified or fabricated their research at least once [9]. However, academia faces misconduct beyond fraud. Workplace sexual harassment complaints comprised 9.8% of all 2018–2021 charges received by the US Equal Employment Opportunity Commission, surpassing 7.7% from 2014 to 2017 [10]. Women in academia, specifically, report the second‐highest rate of sexual harassment following the military [11]. The Association of American Universities reported in 2019 that almost 26% of women undergraduates suffered from non-consensual sexual contact [12]. The scale of the problem might be even more alarming, as a large part of sexual misconduct in academia goes unreported [13]. The above transgressions in academia may impact the perception of the moral and competence traits of the culprit in different ways. Thus, we aimed to investigate the social perception of fraud and sexual harassment through the lens of the Moral Primacy Model [14].

### The social cognition of morality and competence

The morality of a person is pivotal in shaping our opinion of them. As established by the Moral Primacy Model (MPM), morality dominates each stage of impression formation and is central to our perception of others [14]. Together with competence, these two traits account for 82% of the variance in global impressions of others [15]. However, they impact these impressions differently. Perceptions of one’s morality resemble one’s moral character [16] and, more importantly, carry significant social consequences. First, judgments of moral character influence whether individuals tend to avoid or approach others [17]. Second, they impact how trustworthy we are to others [18]. Finally, they can impact life-or-death decisions, as perceptions of untrustworthiness predict convicts’ death (vs. life) sentences [19].

The evidence suggests that morality is usually more important than competence in impression formation. However, this could be context dependent. For instance, a politician receives worse job evaluations (i.e., lower competence ratings) when involved in tax evasion than infidelity [20]. Correspondingly, targets who cheated on lab tasks were judged less competent than control or moral targets [21]. Alternatively, individuals with a greater capacity to harm others may be seen as more competent [22]. Together, research suggests that sexual harassment should be perceived as more harmful than fraud. Since harm is central to moral perception [23], sexual harassers should be judged as less moral than fraudsters.

Consequently, people should demand more punishment for sexual harassers than for fraudsters. Regarding competence judgments, people could judge sexual harassers as more competent than fraudsters because of their harm capacity [22]. Alternatively, previous research suggests that immoral individuals are seen as less competent [21]. However, gender differences might further impact these effects.

### Gender differences in the social perception of sexual harassers

According to Office for National Statistics [24], UK women are more likely than men to experience rape, with almost four times as many women as men victims. Most victims who had experienced rape or assault reported that the perpetrator(s) were men [24]. While harassment is not solely a women’s issue, men-to-women harassment is its most prevalent form [25]. This is reflected in differences in the perceptions of sexual harassers. Firstly, men blame the perpetrator less than women [26], and men’s greater blaming of the women victim is explained by their greater empathy for the men perpetrator [27]. Specifically in academia, women found professors accused of sexual harassment more guilty and assigned more severe punishments than men [28].

The gender differences regarding social and moral perceptions of sexual harassers could be explained through the lenses of egocentric ethics [29]. Specifically, moral judgments are frequently biased by an egocentric perspective, which is easily and automatically accessible but challenging to overcome [30]. Consequently, people perceive others similar to them as morally superior [31]. This effect could be triggered by the similarity of socio-political beliefs [32], personal preferences [33], or group interests [34]. For example, people are more lenient in their judgments toward alleged sexual harassers who share their political views compared to those who hold opposing stances [35]. Therefore, we argue that men would judge men who commit sexual harassment more leniently and demand less punishment for them than women because of a biased perception of their moral character.

### Overview of the present studies

How are sexual harassers and fraudsters judged, depending on the context (academic vs. business) of their actions? We conducted four experiments with UK and US samples to answer that question. We presented participants with a fake newspaper article that described a case of sexual harassment or fraud by a professor in an academic context or a businessman in a business context. We measured how participants perceived these two offenders’ moral character and competence and how much they should be punished for their actions (Study 1). In addition, we measure participants’ perception of harm done by offenders (Study 2). We further examined whether participants’ moral character judgments could be explained by moral rationalization (Study 3a). Finally, we tested if participants used the moral decoupling technique to separate their moral character judgments from competence judgments (Study 3b). With that, we examined gender differences in social perception (morality and competence) of sexual and fraud transgressors and the consequences of their actions (harm and punishment). This allowed us to examine the mechanisms explaining said differences (rationalization and moral decoupling). Table 1 presents a comparison of all studies.

**Table 1:** Comparison of the study designs.

	Study design				
Study	Context	Offence	Probe	Participant’s Gender	Measures
Study 1	Academic vs. Business	Fraud vs. Sexual Harassment	Suspected	Men / Women	Moral character, Competence, Punishment
Study 2	Academic vs. Business	Fraud vs. Sexual Harassment	Guilty vs. Suspected	Men / Women	Moral character, Competence, Punishment, Harm
Study 3a	Academic vs. Business	Fraud vs. Sexual Harassment	Guilty	Men / Women	Morality, Moral Rationalization
Study 3b	Academic vs. Business	Fraud vs. Sexual Harassment	Guilty	Men / Women	Competence, Moral Decoupling

The main goal of the current research program was to expand our knowledge of the understudied perception of sexual and financial offenders and how the context of their actions shapes social judgments about them. Understanding when and why people differ in their moral judgments and willingness to punish wrongdoers is crucial for advancing theoretical knowledge and ensuring workplace safety. By identifying the mechanisms that shape people’s moral and social judgments, we can better explain discrepancies in how people evaluate the same offenses. This can guide efforts to promote fair and consistent decision-making and foster more constructive resolutions in workplace settings.

## Study 1

In Study 1, we sought to investigate how people perceive the moral and competence traits of sexual harassers and fraudsters in academic and business contexts. We presented participants with an alleged case of sexual harassment or fraud in the academic or business context. We measured participants’ perceptions of the offender’s morality and competence traits and to what extent they should be punished. We assumed that moral character judgments would explain the magnitude of punishment. In other words, we predicted that the more offenders are perceived as immoral, the more punishment they deserve.

Since harm is central to moral judgment [23, 36], we hypothesized that the sexual harasser would be judged as less moral than the supposed fraudster because sexual harassment should be perceived as more harmful than fraud (H1). Lower moral character judgments for sexual harassers than for fraudsters should lead participants to higher punishment judgments for the former than the latter (H2). However, the effect of moral character judgments on punishment should be moderated by the participants’ gender. According to the theory of egocentric ethics [29], egocentrism biases moral judgments [30]. Thus, women should perceive sexual harassers as more immoral and demand more punishment for them than men. The same should be valid for fraudsters since both perpetrators are presented as men (H3). Finally, we hypothesized that the supposed sexual harasser would be judged more competent than the supposed fraudster (H4) as individuals with greater harm capacity may be seen as more competent [22]. However, it is also possible that both offenders would be judged as low in competence since immoral targets are seen as less competent than moral targets [21].

### Method

In this article, we report all measures and any data exclusions. Any additional measures not included in the primary analyses are reported in the Supplement. The reported studies were approved by the ethical committee of SWPS University (Ethics Clearance ID: WKE/S 2020/23/I/74). All participants provided informed consent electronically by selecting the consent form at the beginning of each study. All raw data files, analysis scripts, and materials used in this article are available for download from the Open Science Framework (OSF; Data are available at https://osf.io/zj9gr/).

#### Participants and procedure

We did not use power analysis for sample size estimation but instead used the rule of thumb and aimed to recruit 200 participants for each condition. In the end, we managed to recruit 805 participants from the UK using the Prolific Academic in April 2020. We excluded 9 participants who did not pass the attention check. This resulted in a total sample of 796 participants (398 women; mean age = 35.14 years, *SD* = 11.28). Based on a sensitivity power analysis conducted with G*Power [37], this sample size provides a power of 0.80 to detect an effect size of *f* = 0.10.

Participants read a mock newspaper article in one of four versions, correspondingly with the study design: 2 (context: academia vs. business) x 2 (act: fraud vs sexual harassment). The article described a well-known businessman or scientist, Gregory Inma, who allegedly fabricated data for a research grant or harassed women. The perpetrator in the article denied the allegations, and an investigation was ongoing (see the Supplement for full text). Next, participants were asked about the perpetrator’s moral character, competence, and appropriate punishment for their actions.

### Measures

**Moral character judgments** were measured using five items adjusted from Abele et al. [38]: “Gregory Inma is just,” “Gregory Inma is honest,” “Gregory Inma is moral,” “Gregory Inma is trustworthy,” and “Gregory Inma is reliable.” Participants indicated to what extent they agreed with each statement using a scale from 1 = *strongly disagree* to 7 = *strongly agree* (α = .90, *M* = 2.43, *SD* = 1.08).

**Punishment judgments** were measured with seven items: “Gregory Inma should be suspended from his job”, “Gregory Inma’s employment contract should be terminated by mutual agreement”, “Gregory Inma’s employment contract should be terminated without prior notice”, “Gregory Inma should resign from his current post.”, “Gregory Inma should be prohibited from practicing his profession”, “Gregory Inma should pay a fine”, and “Case of Gregory Inma should be prosecuted”. Participants responded on a scale from 1 = *strongly disagree* to 7 = *strongly agree* (α = .84, *M* = 5.13, *SD* = 1.23).

**Competence judgments** of the target were measured using five items adjusted by Abele et al. [38]: “Gregory Inma is efficient,” “Gregory Inma is capable,” “Gregory Inma is competent,” “Gregory Inma is intelligent,” and “Gregory Inma is bright.” Participants indicated to what extent they agreed with each statement using a scale from 1 = *strongly disagree* to 7 = *strongly agree* (α = .89, *M* = 4.33, *SD* = 1.23).

### Results

**Table 2 presents zero-order correlations.** Moral character and competence judgments were positively correlated, and punishment was negatively correlated with both competence and moral character judgments.

**Table 2:** Table of correlations for main variables (Study 1).

Variables	1	2	3
1. Morality	-		
2. Competence	.47***	-	
3. Punishment	-.61***	-.32***	-

Note.

* p < .05

** p < .01

*** p < .001.

#### Moral character judgments

**Fig 1:**
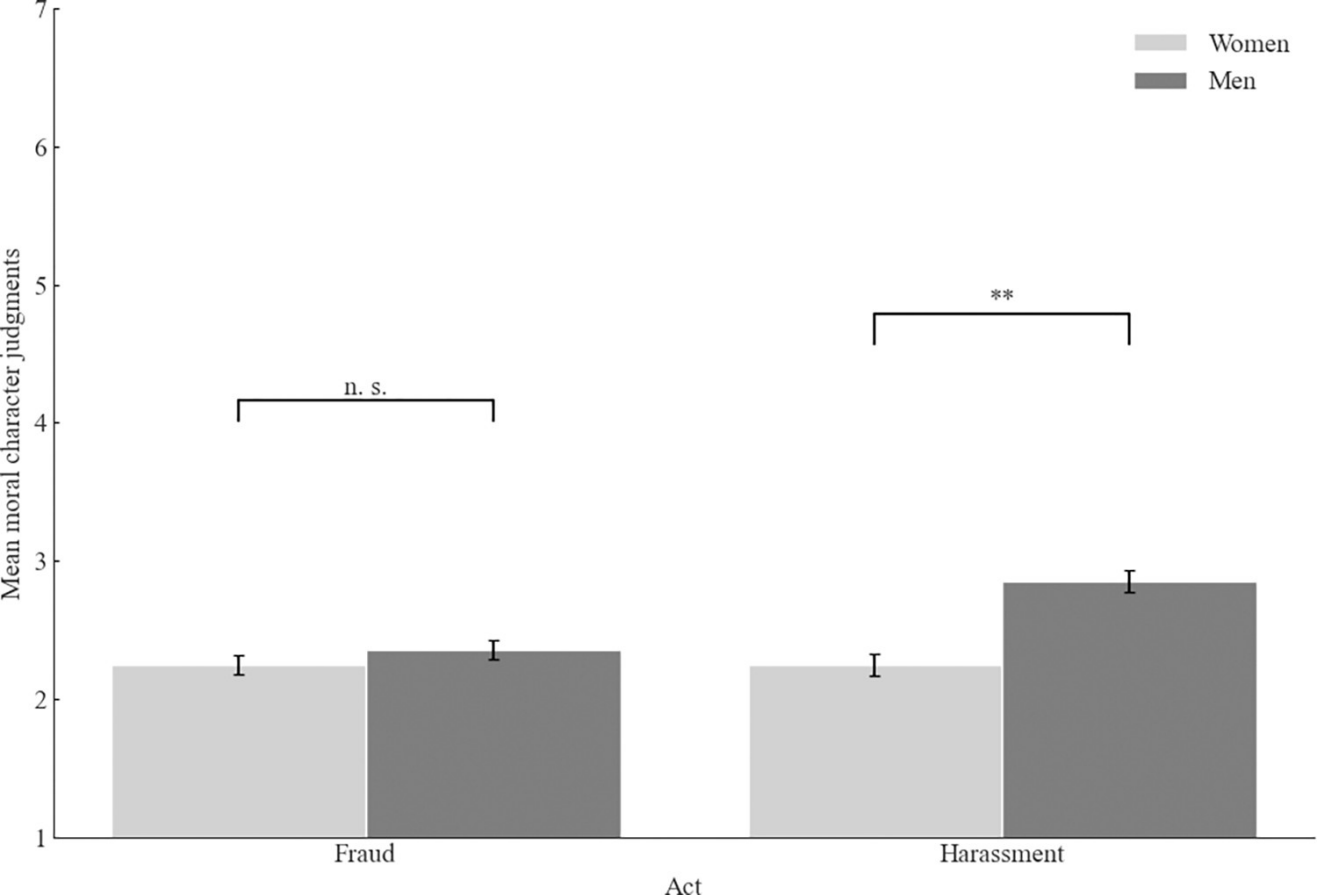
Mean moral character judgments as a function of participant’s gender and act (Study 1). *Note*. Higher scores indicate better moral character judgments. The error bars represent one standard error.

To test our predictions, we have performed a 2 (context: academia vs. business) x 2 (act: fraud vs. sexual harassment) x 2 (participants’ gender: men vs. women) between-participants ANOVA. This analysis yielded a significant main effect of gender. Women made harsher moral judgments than men (*M* = 2.25, *SD* = 0.99 vs. *M* = 2.62, *SD* = 1.13), *F*(1, 788) = 22.51, *p* < .001, ω^2^_p_ = .03, 95% CI [.01, .05]. Moreover, the main effect of the act was also significant, with the supposed sexual harasser judged as less immoral (*M* = 2.57, *SD* = 1.11) than the supposed fraudster (*M* = 2.30, *SD* = 1.04), *F*(1, 788) = 10.92, *p* < .001, ω^2^_p_ = .01 95% CI [.00, .03]. The last significant effect was the interaction between the act and the participant’s gender, *F*(1, 788) = 10.90, *p =* .001, ω^2^_p_ = .01, 95% CI [.00, .03] (see Fig 1). Men judged the supposed sexual harasser as less immoral than women (*M* = 2.85, *SD* = 1.13 vs. *M* = 2.25, *SD* = 0.99), *t*(396.95) = 5.67, *p* < .001, *d =* 0.56 95% CI [.36, .76]. For the supposed fraudster, the difference between men and women was nonsignificant, *t*(395) = 1.02, *p* = .307.

#### Punishment judgments

**Fig 2:**
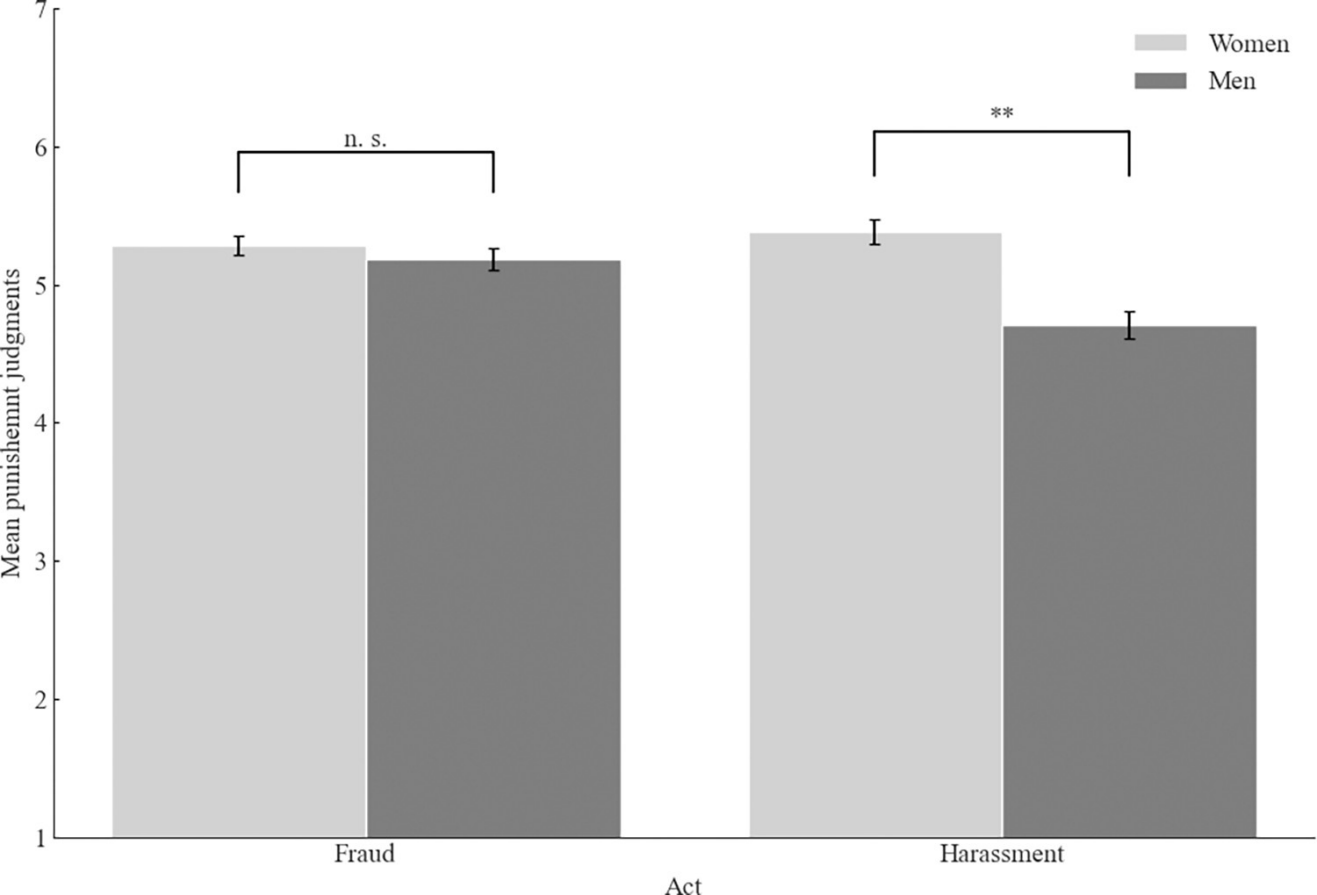
Mean punishment judgments as a function of participant’s gender and act (Study 1). *Note*. Higher scores indicate more severe punishment judgments. The error bars represent one standard error.

The 2 (context: academia vs. business) x 2 (act: fraud vs. sexual harassment) x 2 (participants’ gender: men vs. women) between-participants ANOVA yielded the main effect of the act, *F*(1, 788) = 4.85, *p* = .028, ω^2^_p_ = .00, 95% CI [.00, .02], with the supposed sexual harasser judged as deserving less punishment (*M* = 5.03, *SD* = 1.33) than the supposed fraudster (*M* = 5.24, *SD* = 1.11). The main effect of the participants’ gender was also significant, *F*(1, 788) = 20.32, *p* < .001, ω^2^_p_ = .02, 95% CI [.01, .05]. Women demanded more punishment (*M* = 5.33, *SD* = 1.07) than men (*M* = 5.13, *SD* = 1.23). Further, we found the interaction between the act and participants’ gender, *F*(1, 788) = 10.69, *p* = .001, ω^2^_p_ = .01, 95% CI [.00, .03] (Fig 2). For the supposed sexual harasser, men demanded less punishment than women (*M* = 4.72, *SD* = 1.44 vs *M* = 5.38, *SD* = 1.10, *t*(388.25) = 5.21, *p* < .001, *d =* 0.51, 95% CI [.32, .71]. There was no difference in women’s and men’s punishment judgments for the supposed fraudster, *t*(395) = .92, *p* = .358.

Finally, the interaction between the context, act, and gender was also significant, *F*(1, 788) = 5.01, *p* = .026, ω^2^_p_ = .01, 95% CI [.00, .02], (see Supplement for more details). In the business context, there was no difference between the supposed sexual harasser and the supposed fraudster in punishment judgments for both men *t*(194.16) = 1.74, *p* = .083, and women *t*(196) = .95, *p* = .343. However, gender differences emerged in the academic context. Precisely, men judged that the supposed fraudster deserves more severe punishment than the supposed sexual harasser (*M* = 5.21, *SD* = 1.21 vs. *M* = 4.59, *SD* = 1.42), *t*(196) = 3.25, *p* = .001, *d =* 0.46 95% CI [.18, .75]. Conversely, women judged that the supposed sexual harasser deserves severe punishment than the supposed fraudster (*M* = 5.45, *SD* = 1.05 vs. *M* = 5.13, *SD* = 1.11), *t*(198) = 2.13, *p* = .035, *d =* 0.30 95% CI [.02, .58].

#### Competence judgments

**Fig 3:**
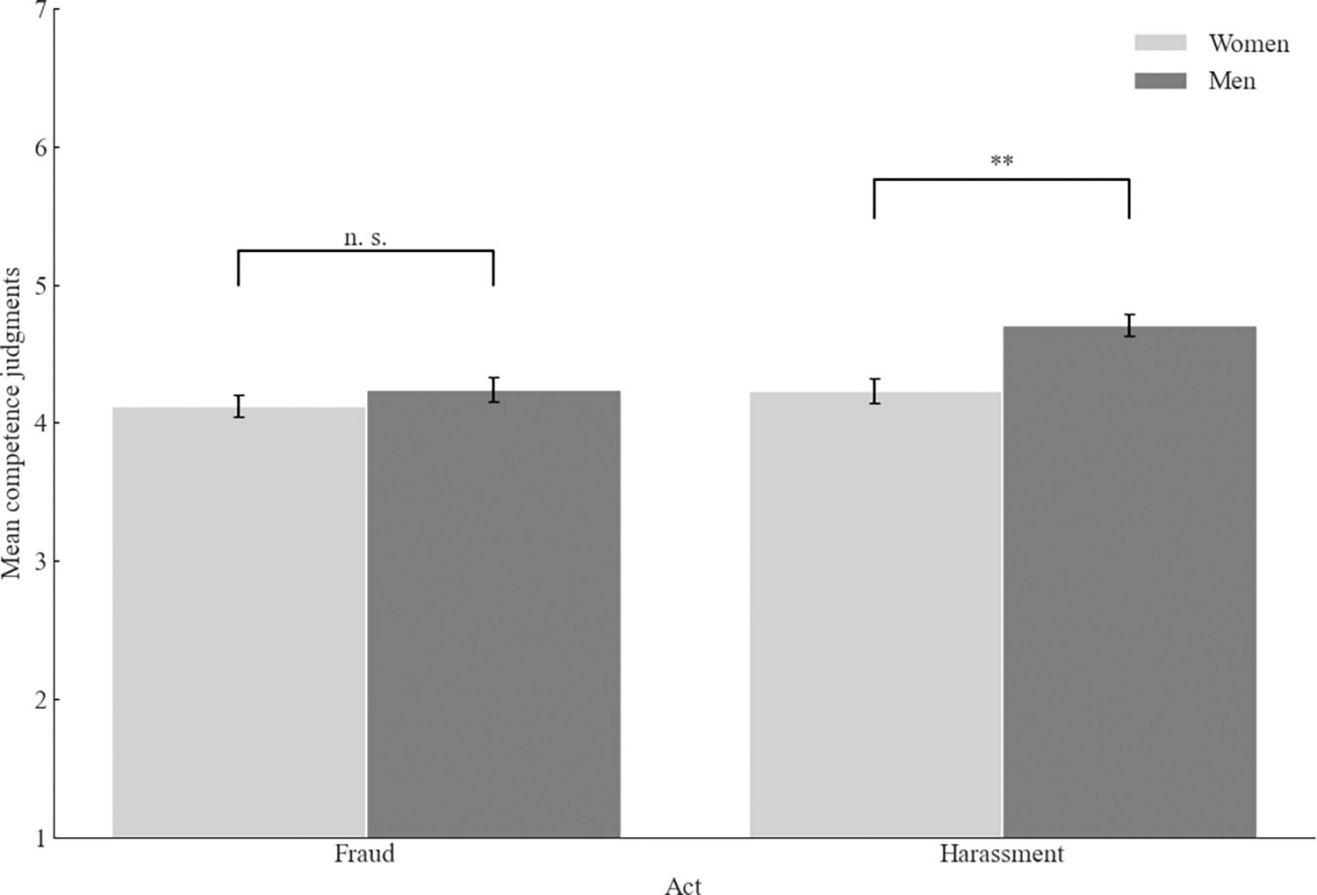
Mean competence judgments as a function of participant’s gender and act. *Note*. Higher scores indicate better competence judgments. The error bars represent one standard error.

We have performed a 2 (context: academia vs. business) x 2 (act: fraud vs. sexual harassment) x 2 (participants’ gender: men vs. women) between-participants ANOVA. First, competence judgments were higher for the academic context than business (*M* = 4.56, *SD* = 1.22 vs. *M* = 4.09, *SD* = 1.21, *F*(1, 788) = 29.72, *p* < .001, ω^2^_p_ = .03, 95% CI [.01, .06]. Second, the supposed sexual harasser was judged as more competent than the supposed fraudster (*M* = 4.48, *SD* = 1.26 vs. *M* = 4.18, *SD* = 1.19, *F*(1, 788) = 11.74, *p* < .001, ω^2^_p_ = .01, 95% CI [.00, .03]. Finally, men’s competence judgments were higher than women’s (*M* = 4.49, *SD* = 1.23 vs. *M* = 4.17, *SD* = 1.22, *F*(1, 788) = 12.77, *p* < .001, ω^2^_p_ = .01, 95% CI [.00, .04]. Moreover, the interaction between the act and participants’ gender was also significant, *F*(1, 788) = 4.22, *p* = .040, ω^2^_p_ = .00, 95% CI [.00, .02] (see Fig 3). Men judged the supposed sexual harasser as more competent than women (*M*, = 4.71, *SD* = 1.20 vs. *M* = 4.23, *SD* = 1.27, *t*(397) = 3.87, *p* < .001, *d =* 0.39 95% CI [.19, .57]. For the supposed fraudster, the difference between men and women was insignificant, *t*(395) = 1.03, *p* = .302.

Lastly, we found the interaction between the context and the act, *F*(1, 788) = 4.55, *p* = .033, ω^2^_p_ = .00, 95% CI [.00, .02]. For the academic context, the supposed sexual harasser was judged as more competent than the supposed fraudster (*M* = 4.81, *SD* = 1.15 vs. *M* = 4.32, *SD* = 1.24, *t*(396) = 4.08, *p* < .001, *d =* 0.41 95% CI [.21, .61]. The business context’s difference was insignificant, *t*(395) = 1.02, *p* = .306.

#### Moderated mediation analysis

**Fig 4:**
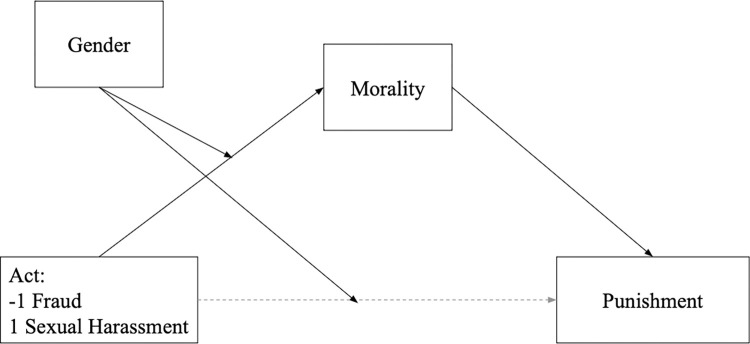
Model 8 for the moderated mediation of the act’s effect on punishment intentions through moral judgments, moderated by gender.

To test whether moral judgments mediate the punishment judgments differently for participants’ gender, we run a moderated mediation. We used Model 8 in PROCESS macro proposed by Hayes [39] (Fig 4) because gender moderated the morality and punishment judgments for the supposed sexual harasser and fraudster. Therefore, we entered the act (sexual harassment vs. fraud) as an independent variable, the moral judgments as a mediator, and the punishment judgments as a dependent variable, with gender as a moderator of the link between the mediator and the dependent variable.

The conditional direct effect of the act on the punishment was moderated by gender, *B* = 0.17, *SE* = 0.05, 95% CI = [0.07, 0.27]. The indirect effect of the act on the punishment, through morality, was significant for men, *B* = -0.17, *SE* = 0.04, 95% CI = [-0.24, -0.09], but non-significant for women, *B* = 0.00, *SE* = 0.03, 95% CI = [−0.06, 0.06]. The conditional direct effect of the act on moral judgments was significant for men, *B* = 0.27, *SE* = 0.05, 95% CI = [0.14, 0.35], but non-significant for women, *B* = 0.00, *SE* = 0.05, 95% CI = [−0.10, 0.10]. Therefore, this result confirms that punishment judgments were explained by the perception of the offender’s morality. However, this mechanism was valid only for men.

### Discussion

The results of Study 1 confirmed some of the hypotheses. To our surprise, the supposed sexual harasser was judged as more moral than the supposed fraudster, and participants demanded less punishment for him than for the supposed fraudster. Therefore, H1 and H2 were confirmed but in the opposite direction. However, both these effects were qualified by the participants’ gender. Precisely, men but not women judged the supposed sexual harasser leniently and demanded less punishment for him. Importantly, moral character judgments explained less severe punishment judgments for men but not for women, confirming H3. In addition, we found that men, in contrast to women, showed higher moral character and less severe punishment judgments for the supposed sexual harasser in the academic but not in the business context. Finally, the supposed sexual harasser was judged more competent than the supposed fraudster, which confirms our assumptions of H4. However, this effect was again qualified by the participant’s gender and the context. Men but not women, perceived the supposed sexual harasser as more competent than the supposed fraudster and only in the academic context.

Results of Study 1 suggest that sexual harassers are perceived as less immoral than supposed fraudsters and deserve less punishment only by men and only in the academic context. However, a severe limitation of Study 1 was that the presented offenders were only accused, not proven guilty. This could impact the believability of the act and, consequently, moral and punishment judgments. Moreover, we assumed that sexual harassment should be perceived as more harmful than fraud, but we did not measure harm perception. Thus, we addressed these limitations in Study 2.

## Study 2

In Study 2, we sought to replicate, generalize, and extend the effects found in Study 1. Therefore, we first used the sample from the UK instead of the US. Second, we added another condition in which we manipulated whether the target was accused or found guilty of sexual harassment or fraud to investigate if this factor could impact the results found in Study 1. Finally, we measured the perception of harm to clarify the assumption that sexual harassment should be seen as more harmful than fraud. The remaining parts of Study 2 were identical to Study 1. In addition to previous predictions, we assumed the guilty transgressor would be judged less moral than the suspected one (H5).

### Method

#### Participants and procedure

The main effect of the act for moral character judgments in Study 1 was *f* = 0.13. Using the G*Power calculator [37], we estimated the target sample size to replicate this effect as *N* = 253 for each condition (assuming a power of 0.80, two-tailed). Therefore, the target sample for Study 2 was estimated at 2024 participants. In the end, using Prolific Academic, we recruited 1507 participants. Data was collected between the May and June 2020. We excluded 11 participants who did not pass the attention check, leaving the final sample of 1496 participants (752 women; mean age = 34.95 years, *SD* = 12.84) from the US. Based on a sensitivity power analysis, this sample size provides a power of 0.80 to detect an effect size of *f* = 0.07.

In study 2, participants read a mock newspaper article in one of eight versions in line with the study design: 2 (context: academia vs. business) x 2 (act: fraud vs. sexual harassment) x 2 (probe: suspected vs. guilty). Therefore, in contrast to Study 1, participants read an additional four versions of the article (see the Supplement for full text) where the target (fraud or sexual harassment) was found guilty of the charge either in the academic or business context. After reading the article, participants were asked to make morality, punishment, and competence judgments, as in Study 1, and judgments regarding harm.

### Measures

**Moral character judgments** were measured as in Study 1 (⍺ = .90, *M* = 2.11, *SD* = 1.08).

**Punishment judgments** were measured as in Study 1 (α = .81, *M* = 5.56, *SD* = 1.12).

**Competence judgments** were measured as in Study 1 (α = .91, *M* = 4.21, *SD* = 1.41).

**Harm** was measured with a single item, “How harmful [i.e., involving physical and/or emotional suffering] were the actions of Gregory Inma?”. Participants rated how they perceived Gregory Inma’s actions on a scale from 1 = *not at all* to 7 = *very* (*M* = 5.68, *SD* = 1.42).

### Results

**Zero-order correlations** are presented in Table 3. Morality judgments were positively correlated with competence judgments. There was a negative relationship between moral judgments and punishment and harm perception. Punishment was positively correlated with the perceived harm of the offence.

**Table 3:** Table of correlations for main variables (Study 2).

Variables	1	2	3	4
1. Morality	-			
2. Competence	.45***	-		
3. Punishment	-.56***	-.28***	-	
4. Harm	-.33***	-.11***	.41***	-

Note.

* p < .05

** p < .01

*** p < .001.

#### Moral character judgments

We have performed a 2 (context: academia vs. business) x 2 (act: fraud vs. sexual harassment) x 2 (probe: suspected vs. guilty) x 2 (participants’ gender: men vs. women) between-participants ANOVA. Similar to Study 1, we found that women made harsher moral judgments than men (*M* = 1.96, *SD* = 0.98 vs. *M* = 2.26, *SD* = 1.14, *F*(1, 1478) = 31.89, *p* < .001, ω^2^_p_ = .02, 95% CI [.01, .04]. Again, we found the main effect of the act, *F*(1, 1478) = 14.78, *p* < .001, ω^2^_p_ = .01, 95% CI [.00, .02], where the sexual harasser was seen as less immoral (*M* = 2.21, *SD* = 1.11) than the fraudster (*M* = 2.00, *SD* = 1.02). We also found that the suspected target was judged as less immoral than the guilty one, confirming the hypothesis (*M* = 2.31, *SD* = 1.11 vs. *M* = 1.92, *SD* = 1.00), *F*(1, 1478) = 52.59, *p* < .001, ω^2^_p_ = .03, 95% CI [.02, .05].

**Fig 5:**
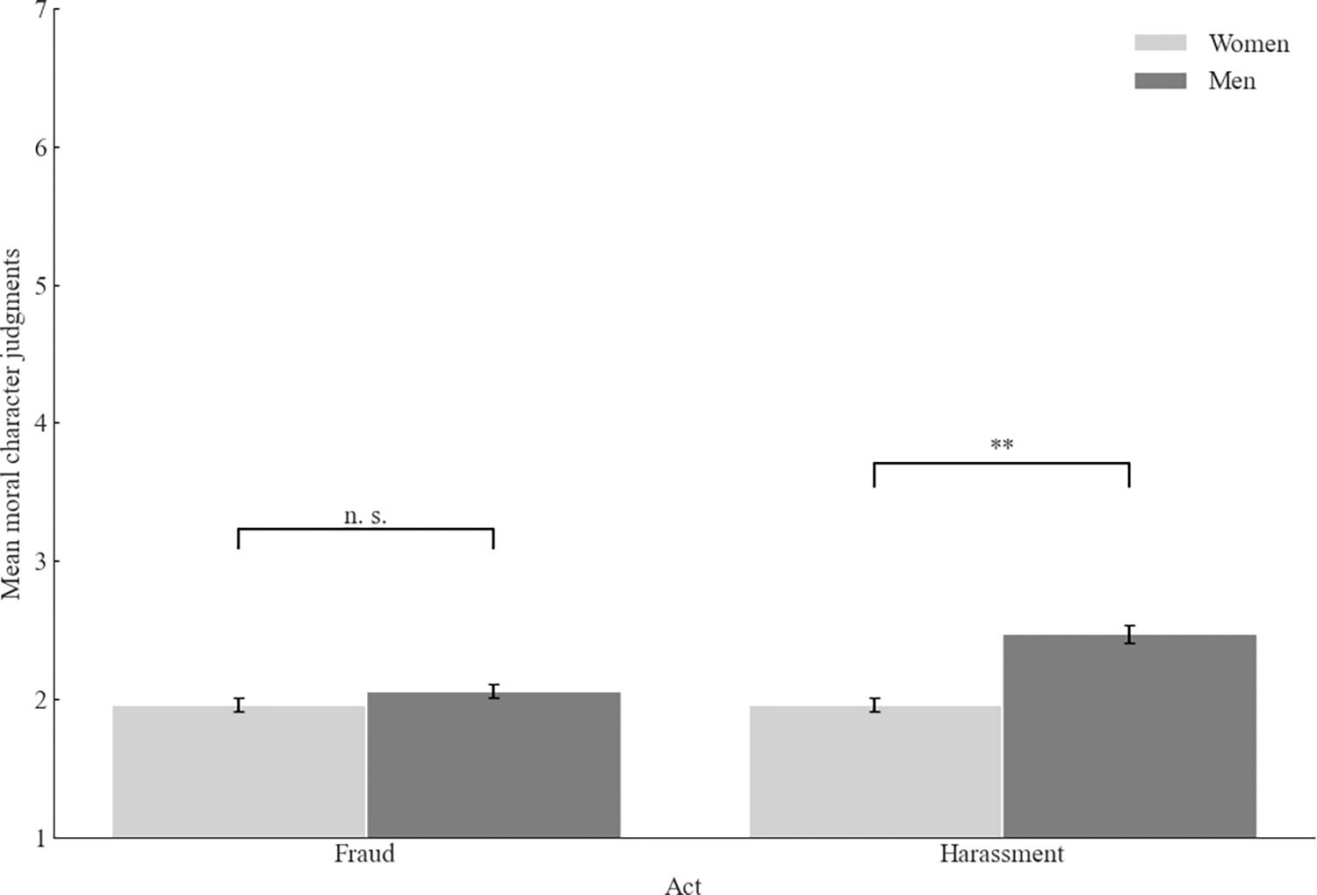
Mean moral character judgments as a function of participant’s gender and act (Study 2). *Note*. Higher scores indicate better moral character judgments. The error bars represent one standard error.

Replicating the results of Study 1, we found the interaction between the act and the participant’s gender *F*(1,1478) = 12.94, *p <* .001, ω^2^_p_ = .01, 95% CI [.00, .02] (Fig 5). Again, men judged the sexual harasser as less immoral than women (*M* = 2.47, *SD* = 1.19 vs. *M* = 1.96, *SD* = 0.97), *t*(712.857) = 6.37, *p* < .001, *d =* 0.47 95% CI [.32, .61]. For the fraudster, the difference between men and women was insignificant, *t*(740) = 1.35, *p* = .177. Finally, we found the interaction between the act and the probe, *F*(1, 1478) = 8.97, *p =* .003, ω^2^_p_ = .01, 95% CI [.00, .02]. Corroborating the result of Study 1, the suspected sexual harasser was judged as less immoral than the alleged fraudster (*M* = 2.49, *SD* = 1.15 vs. *M* = 2.12, *SD* = 1.04), *t*(734.550) = 4.53, *p* < .001, *d =* 0.33 95% CI [.19, .48]. However, this difference was eliminated when the target was found guilty of the charge, with no difference between the sexual harasser and the fraudster, *t*(752) = .54, *p* = .593.

#### Punishment judgments

**Fig 6:**
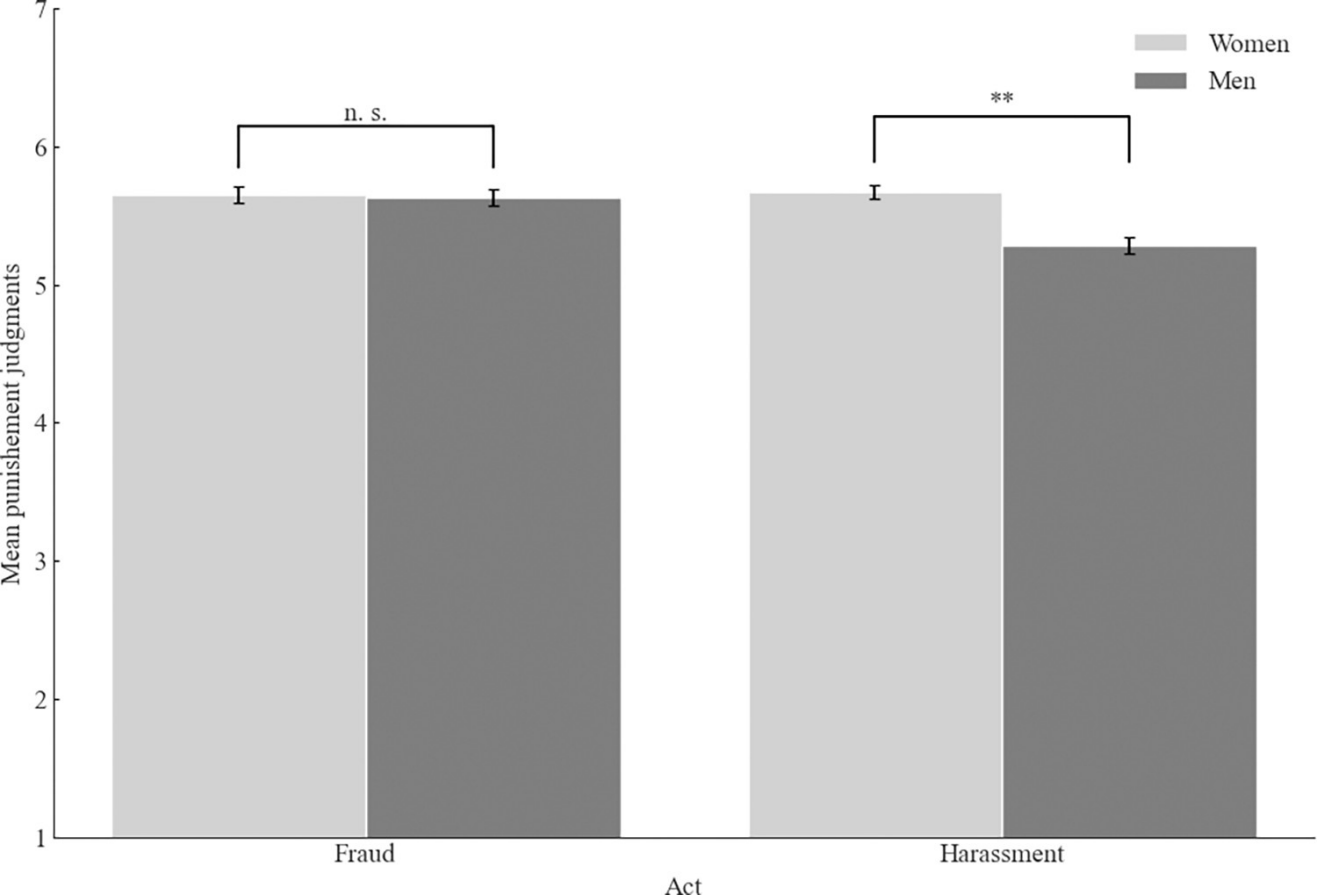
Mean punishment judgments as a function of participant’s gender and act (Study 2). *Note*. Higher scores indicate more severe punishment judgments. The error bars represent one standard error.

As in Study 1, we found the main effect of the act, *F*(1, 1478) = 8.71, *p* = .003, ω^2^_p_ = .01, 95% CI [.00, .01] and the main effect of the gender, *F*(1, 1478) = 12.24, *p* < .001, ω^2^_p_ = .01, 95% CI [.00, .02]. Moreover, we also found the main effect of the probe, *F*(1, 1478) = 36.61, *p* < .001, ω^2^_p_ = .02, 95% CI [.01, .04], with the guilty target deserving more punishment than the suspected target (*M* = 5.73, *SD* = 1.04 vs. *M* = 5.38, *SD* = 1.17). More importantly, we found the interaction between the act and participants’ gender, *F*(1, 1478) = 9.20, *p* = .002, ω^2^_p_ = .01, 95% CI [.00, .02], replicating results of Study 1 (Fig 6). Again, women demanded more punishment for the sexual harasser than men (*M* = 5.67, *SD* = 1.14 vs. *M* = 5.28, *SD* = 1.17, *t*(750) = 4.57, *p* < .001, *d =* 0.33 95% CI [.19, .48]. There was no difference in women’s and men’s punishment judgments for the fraudster, *t*(740) = .26, *p* = .798.

The interaction between the act, the participants’ gender and the probe was nonsignificant, *F*(1, 1478) = 1.44, *p* = .230. However, we found the interaction between the act and the probe, *F*(1, 1478) = 8.24, *p* = .004, ω^2^_p_ = .00, 95% CI [.00, .01]. Specifically, when the target was accused of sexual harassment, participants demanded less punishment than when the target was accused of fraud (*M* = 5.23, *SD* = 1.25 vs. *M* = 5.55, *SD* = 1.06), *t*(725.547) = 3.91, *p* < .001, *d =* 0.29 95% CI [.14, .43]. However, when both targets were found guilty, the difference was eliminated: *t*(752) = 0.02, *p* = .986.

#### Competence

We ran a 2 (context: academia vs. business) x 2 (act: fraud vs. sexual harassment) x 2 (probe: suspected vs. guilty) x 2 (participants’ gender: men vs. women) between-participants ANOVA and found the interaction effect for the context and the act, *F*(1, 1478) = 40.28, *p* < .001, ω^2^_p_ = .03, 95% CI [.01, .04]. Replicating the results of Study 1, for the academic context, the sexual harasser was judged as more competent than the fraudster (*M* = 4.78, *SD* = 1.42 vs. *M* = 4.12, *SD* = 1.28, *t*(744.820) = 6.63, *p* < .001, *d =* 0.48 95% CI [.34, .63]. However, in contrast to Study 1, in the business context, the difference was significant, with the sexual harasser being judged as less competent than the fraudster (*M* = 3.85, *SD* = 1.43 vs. *M* = 4.09, *SD* = 1.34), *t*(740) = 2.28, *p* = .023, *d =* 0.1795% CI [.02, .31].

#### Harm

We found the main effect of the act, *F*(1, 1478) = 104.40, *p* < .001, ω^2^_p_ = .06, 95% CI [.04, .09]. The sexual harasser was judged more harmful than the fraud (*M* = 6.05, *SD* = 1.26 vs. *M* = 5.32, *SD* = 1.47). Moreover, we found that regardless of the act, women’s judgments of harm were higher than men’s judgments (*M* = 5.84, *SD* = 1.36 vs. *M* = 5.53, *SD* = 1.45), *F*(1, 1478) = 18.48, *p* < .001, ω^2^_p_ = .01, 95% CI [.00, .02]. Finally, we found only one interaction between the act and the probe, *F*(1, 1478) = 4.36, *p* = .037, ω^2^_p_ = .00, 95% CI [.00, .01]. Specifically, the sexual harassment was more harmful when it was proven than suspected (*M* = 6.17, *SD* = 1.25 vs. *M* = 5.92, *SD* = 1.26), *t*(750) = 2.69, *p* = .007, *d =* 0.20 95% CI [.05, .34]. There was no difference in harm judgments for the fraud between the suspected and guilty conditions, *t*(742) = 0.47, *p* = .640.

#### Moderated mediation analysis

Correspondingly with Study 1, gender moderated morality and punishment judgments. Therefore, we run a moderated mediation Model 8 in PROCESS macro [39]. Replicating the results of Study 1, the indirect effect of the act on the punishment, through morality, was significant for men, *B* = -0.12, *SE* = 0.02, 95% CI = [-0.16, -0.07], but nonsignificant for women, *B* = 0.00, *SE* = 0.02, 95% CI = [−0.04, 0.04]. Moreover, and like Study 1, the conditional direct effect of the act on moral judgments was significant for men, *B* = 0.20, *SE* = 0.04, 95% CI = [0.13, 0.28], but nonsignificant for women, *B* = 0.00, *SE* = 0.04, 95% CI = [−0.07, 0.08].

### Discussion

Study 2 replicated and extended Study 1 using a larger and different sample and analyzing additional factors such as proven guilty of offenders and harm perception. As in Study 1, the sexual harasser was judged as less immoral than the fraudster, but only by men, and only men demanded less punishment for their actions. Again, we confirmed that moral judgments were the driving force behind the punishment judgments and that this effect was true for men but not women. Finally, we again showed that the sexual harasser was judged more competent than the fraudster, but only in an academic context.

Addressing the limitations of Study 1, we compared accused transgressors to guilty ones. Confirming our hypothesis, the guilty transgressor was judged less moral than the suspected one. Further, we found that proof of guilt (vs. being suspected) reduced the leniency in moral judgments of the sexual harasser compared to the fraudster. However, it is important to stress that even in the face of information about guilt, the sexual harasser was not judged as less moral than the fraudster but equally immoral. The same was valid for punishment judgments. We also confirmed that sexual harassment was perceived as more harmful than fraud, but this effect was not moderated by gender.

The results of Study 1 and Study 2 suggest that men make more biased moral judgments of sexual harassers compared to women, leading them to conclude that sexual harassers deserve less punishment than fraudsters. Moreover, sexual harassers were seen as less immoral and more competent than fraudsters, but only in the academic context, which additionally suggests that social cognition of sexual harassment is context-dependent and differs between academic and business contexts. This cannot be explained by the perception of harm (see Gray et al. [22]), as sexual harassment was perceived as more harmful than fraud independently of the participants’ gender.

Hence, in the following two studies, we sought to understand why sexual harassers are perceived differently by men and women regarding moral character judgments and differently in academic and business contexts when their competence is judged. Specifically, we investigated moral rationalization (Study 3a) as a potential psychological mechanism explaining moral character judgments and moral decoupling (Study 3b), explaining competence judgments.

## Study 3a

When the concept of a sexual harasser is made salient, men’s moral identity might be threatened as men are more frequently reported as perpetrators of sexual harassment [24]. This threat may produce tension between a desire to maintain a positive moral view about themselves and a social anticipation to condemn the harasser. Men may solve this tension through moral rationalization.

Moral rationalization could be defined as the mechanism by which immoral actions are reconstructed to view them as less immoral [40]. This is possible because moral judgments are complex, while moral dilemmas leave room for ambiguity, allowing arguments or motivation to impact the direction of moral reasoning [40]. Hence, in Study 3a, we test whether moral rationalization could explain why men judge sexual harassers as less immoral than fraudsters. We aimed to replicate, as in Studies 1 and 2, that men but not women would judge the sexual harasser as less immoral than the fraudster. Moreover, we formed new prediction that men, but not women, would use moral rationalization to justify the act of sexual harassment, and this could explain the gender differences in moral character judgments (H6).

### Method

#### Participants and procedure

The average main effect of the act for moral character judgments for Study 1 and Study 2 was *f* = 0.11. We estimated the target sample size to replicate this effect as *N* = 1028 (assuming the power of 0.80, one-tailed). Using Prolific Academic, we recruited 924 US participants in August 2020. We removed 18 participants for not passing the attention check, resulting in the final sample of 906 participants (453 women; mean age = 33.94 years, *SD* = 10.84). Based on a sensitivity power analysis, this sample size provides a power of 0.80 to detect an effect size of *f* = 0.09. Participants read the same mock newspaper article as in Study 2 about a sexual harasser or fraudster being found guilty of charges in the academic or business context. Next, they judged the perpetrator’s moral traits and answered questions about moral rationalization.

### Measures

**Moral character judgments** were measured as in Studies 1 and 2 (⍺ = .95, *M* = 1.93, *SD* = 1.27).

**Moral rationalization** was measured with five items adapted from Bhattacharjee et al. [40]. Items from the original scale, such as „People should not be at fault for lying on their taxes because the system is too complicated”, were adjusted to fit the articles in the study for the sexual harassment „People should not be at fault for sexual harassment because the social norms are too complicated” or fraud „People should not be at fault for lying on their grant reports because the system is too complicated” (sexual harassment: ⍺ = .90, *M* = 1.43, *SD* = 1.02; fraud: ⍺ = .90, *M* = 1.72, *SD* = 1.13; see the Supplement for further details).

### Results

#### Moral character judgments

Again, we found the interaction effect between the act and the participants’ gender, *F*(1, 895) = 8.98, *p* = .003, ω^2^_p_ = .01, 95% CI [.00, .02]. Corroborating the results of Studies 1 and 2, men perceived the sexual harasser as less immoral than women, *M* = 2.38, *SD* = 1.67 vs. *M* = 1.39, *SD* = 0.67, *t*(278.89) = 8.33, *p* < .001, *d =* 0.79 95% CI [.59, .98], (see the Supplement for a full analysis).

#### Moral rationalization

We found that men used moral rationalization to more extent than women, *M* = 1.85, *SD* = 1.35 vs. *M* = 1.30, *SD* = 0.62, *F*(1, 895) = 61.95, *p* < .001, ω^2^_p_ = .06, 95% CI [.04, .10]. However, the interaction between the act and participants’ gender was nonsignificant, *F*(1, 895) = .46, *p* = .497 (see the Supplement for a full analysis).

#### Moderated mediation analysis

**Fig 7:**
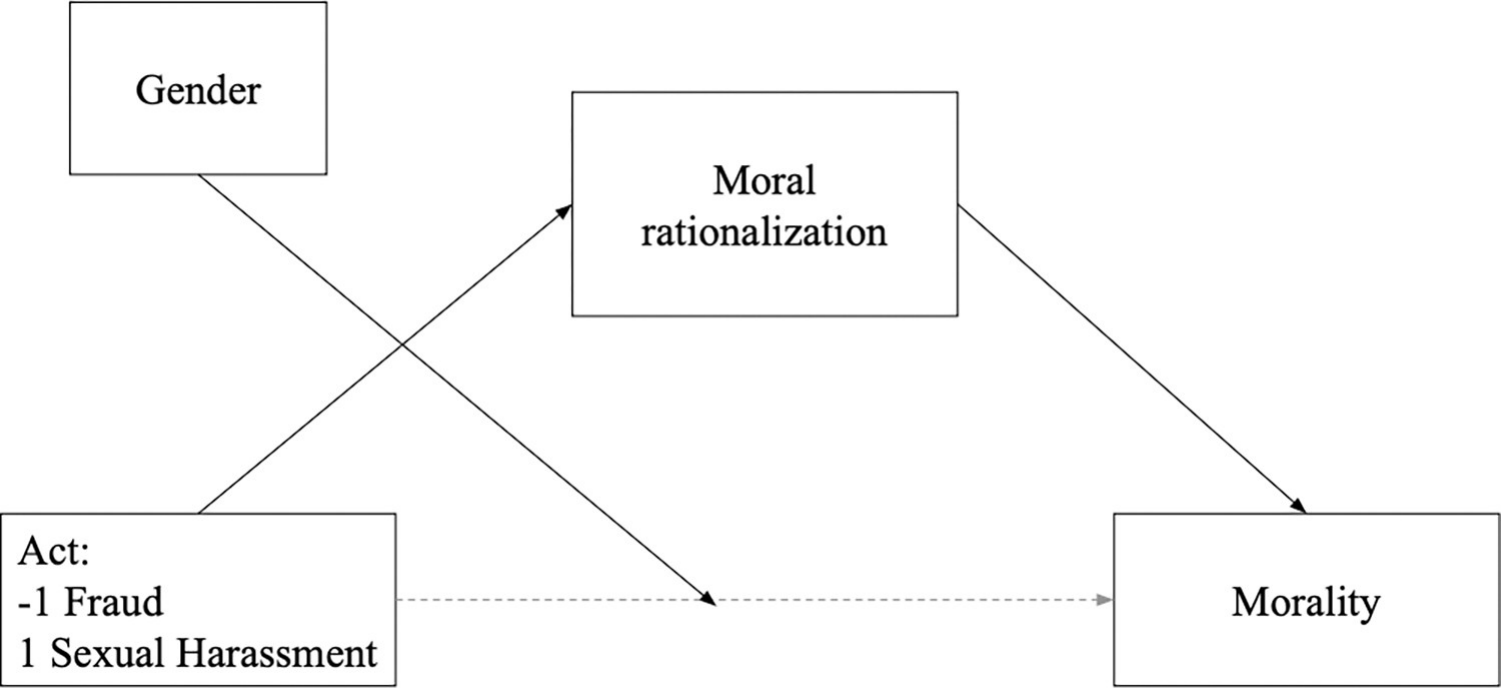
Model 5 for the moderated mediation of the effect of the act on moral judgments through moral rationalization, moderated by gender.

As gender moderated only the morality judgments, we ran a moderated mediation Model 5 in PROCESS macro [39], see Fig 7. The conditional direct effect of the act on morality judgments was moderated by gender, *B* = -0.10, *SE* = 0.03, 95% CI = [-0.16, -0.04] and was significant for men, *B* = 0.17, *SE* = 0.04, 95% CI = [0.09, 0.25], but nonsignificant for women, *B* = -0.03, *SE* = 0.04, 95% CI = [−0.11, 0.05]. The indirect effect of the act on morality, through moral rationalization, was significant but in the opposite direction than predicted, *B* = -0.12, *SE* = 0.03, 95% CI = [-0.18, -0.07]. Overall, these results corroborate findings from Studies 1 and 2 that men, but not women perceive sexual harassers as less immoral than fraudsters. However, the assumption that moral rationalization could explain this effect was not confirmed.

## Study 3b

In Study 1 and Study 2, we found the same pattern of results showing that sexual harassers were judged as more competent but only in the academic and not business context. This cannot be linked to the perception of harm because, in Study 2, harm was negatively correlated with competence. An explanation of this effect could be related to the moral decoupling process. Moral decoupling is a form of moral reasoning that does not condone immoral behavior, allowing people to selectively dissociate judgments of competence from morality. Notably, this process is reduced when the transgression is directly relevant to the performance domain [40].

We assumed that fraud, in contrast to sexual harassment, is directly related to performance. By decoupling morality from competence, people could acknowledge the immorality of the act but still perceive the harasser as competent. Therefore, the moral decoupling process should explain why sexual harassers are seen as more competent than fraudsters in the academic context. We tested our assumptions in Study 3b and hypothesized that participants would use moral decoupling to judge sexual harassers but not fraudsters (H7).

### Method

#### Participants and procedure

In Study 1 and Study 2, the average interaction effect of the act and the context for competence judgments was *f* = 0.13. Therefore, we estimated the target sample size to replicate this effect as *N* = 456 (assuming a power of 0.80, one-tailed). We recruited 501 participants using Prolific Academic and excluded 5 for failing to pass the attention check. The data was collected in the August of 2020. The final sample was 496 US participants (252 women; mean age = 35.49 years, *SD* = 11.07). Based on a sensitivity power analysis, this sample size provides a power of 0.80 to detect an interaction effect size of *f* = 0.13. Participants read the same mock newspaper article as in Study 3a, later judged the target’s competence, and answered questions related to moral decoupling.

### Measures

**Competence judgments** were measured as in Studies 1 and 2 (⍺ = .91, *M* = 4.30, *SD* = 1.44).

**Moral decoupling** was measured with three items adapted from Bhattacharjee et al. [40]: “Personal actions do not change my assessment of Gregory Inma’s competence”, “Judgments of competence should remain separate from judgments of morality”, “Reports of wrongdoing should not affect our view of Gregory Inma’s achievements”. Participants responded on a scale from 1 = *strongly disagree* to 7 = *strongly agree* (⍺ = .81, *M* = 3.40, *SD* = 1.55).

### Results

#### Competence judgments

Corroborating findings of Studies 1 and 2, we found the interaction effect between the act and the context, *F*(1, 488) = 17.43, *p* < .001, ω^2^_p_ = .03, 95% CI [.01, .07]. Further analysis confirmed that in the academic context, the sexual harasser was judged as more competent (*M* = 4.77, *SD* = 1.31) than the fraudster (*M* = 3.98, *SD* = 1.44, *t*(242) = 4.46, *p* < .001, *d =* 0.57 95% CI [.32, .83]. For the business context, this difference was nonsignificant, *t*(250) = 1.65, *p* = .101.

#### Moral decoupling

We found the interaction effect between the act and the context, *F*(1, 488) = 9.86, *p* = .002, ω^2^_p_ = .02, 95% CI [.00, .05]. In the academic context, moral decoupling was higher for the sexual harasser (*M* = 3.92, *SD* = 1.58) than the fraudster (*M* = 3.08, *SD* = 1.49), *t*(242) = 4.25, *p* < .001, *d =* 0.54 95% CI [.29, .80]. For the business context, there was no difference between the perpetrators, *t*(250) = .36, *p* = .722.

#### Moderated mediation analysis

**Fig 8:**
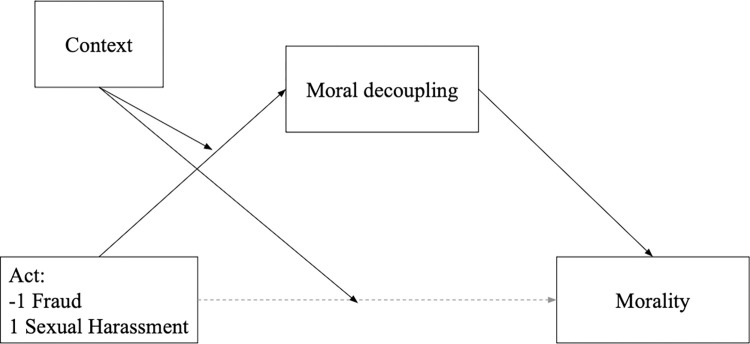
Model 8 for the moderated mediation of the effect of the act on moral judgments through moral decoupling, moderated by context.

As the context moderates the competence judgments and moral decoupling, we run a moderated mediation Model 8 in PROCESS macro [39], see Fig 8. The context moderated the conditional direct effect of the act on competence judgments, *B* = 0.16, *SE* = 0.05, 95% CI = [0.05, 0.26]. It was significant for the academic context, *B* = 0.19, *SE* = 0.08, 95% CI = [0.04, 0.34], but nonsignificant for the business context, *B* = -0.13, *SE* = 0.07, 95% CI = [−0.28, 0.02]. The indirect effect of the act on competence judgments through moral decoupling was significant for the academic context, *B* = 0.21, *SE* = 0.05, 95% CI = [0.11, 0.31], but nonsignificant for the business context, *B* = -0.02, *SE* = 0.05, 95% CI = [-0.11, 0.07].

These results, confirming our hypothesis, demonstrate that in the case of the sexual harasser, but not the fraudster, participants used the moral decoupling process to separate judgments of competence from the target’s morality. As a result, the sexual harasser was judged more competent than the fraudster. Notably, this moral decoupling process was uniquely observed in the academic context and not the business context, accounting for the varying perceptions of harassers versus fraudsters between these environments. This was true for both men and women, as neither act nor context interacted with participants’ gender.

## General discussion

We investigated how people perceive the competence and morality of sexual harassers and fraudsters in business and academic contexts. Across four studies, we showed that the sexual harasser was seen as less immoral than the fraudster (Studies 1 and 2), even though the sexual harasser was seen as causing more harm than the fraudster (Study 2). The participant’s gender drove these differences, as men judged the sexual harasser less immoral and demanded less punishment for sexual harassment than women (Studies 1 and 2). Further, the moral rationalization did not explain the observed gender differences in moral character judgments of the sexual harasser (Study 3a). Finally, in the academic but not in the business context, the sexual harasser was judged as more competent than the fraudster (Studies 1, 2, and 3b). This difference was driven by the moral decoupling process, which allowed participants, regardless of their own gender, to separate competence from moral judgments (Study 3b).

We have advanced the current literature in several ways. First, we showed that men perceived sexual harassers as less immoral than women. Second, we documented that moral character judgments were underpinning mechanisms explaining why men demanded less punishment for sexual harassers than women. Finally, although past studies suggested that immoral actions could be reconstructed to view them as less immoral via the moral rationalization mechanism [41], we did not find evidence for this mechanism in men’s moral character judgments of sexual harassers.

We further expand previous studies, which showed that perception of competence depends on the context [20], by demonstrating that sexual harassers were perceived as more competent than fraudsters in the academic but not in the business context. For the business context, this result corresponds with past research, showing that immoral targets are seen as less competent than moral targets [21]. However, in the academic context, these findings [21] were not confirmed, nor the argument that individuals with greater harm capabilities should be seen as more competent [22]. We aimed to resolve this by showing that both men and women use moral decoupling [40] to separate moral and competence judgments in the academic, not business, context.

### Limitations, implications, and future directions

Our work has some limitations that warrant future research. First, even though our samples represented equally men and women from the UK (Study 1) and the US (Study 2) and were well-powered, we acknowledge that the presented results are limited to people who live in Western, Educated, Industrialized, Rich, and Democratic (WEIRD) countries [42]. Our second limitation is the focus on men perpetrators. For sexual harassment [43] and scientific fraud [44], the typical perpetrator is a man, but women also commit those wrongdoings. Hence, our results cannot be generalized to women perpetrators.

Future research might expand our attempt to understand the psychological processes explaining moral and competence judgments of sexual harassment. Study 3b found that people use the moral decoupling mechanism to separate competence from moral judgments independently from gender differences. However, this was true only for sexual harassers in the academic context. Future studies could explain why the moral decoupling process does not occur in business.

By demonstrating that moral decoupling occurs in the perception of harassing academics but not entrepreneurs, our work might contribute to understanding why the #MeToo movement did not resonate as much in academia as in business. For instance, accusations of data fabrication in academia often lead to swift and decisive actions, such as investigations and removing scientists from their positions [45]. However, when sexual harassment is reported in academic settings people may separate morality from competence. This could explain why a well-known biologist who violated anti-harassment policies was removed from MIT, only to receive significant financial support from the private sector [46].

The work we have done can serve as an initial step towards gaining a better understanding of the variations in people’s perceptions of sexual harassment and fraud in various social settings. This will enable future studies to investigate whether the differences observed in moral decoupling can be mitigated in order to establish a consistent approach to misconduct.

## Conclusion

This research program examines how people judge the moral character and competence traits of sexual harassers and fraudsters in the academic and business context. Men consistently judged sexual harassers as less immoral than women and demanded less punishment for them. These gender differences could not be explained by moral rationalization. At the same time, participants judged sexual harassers as more competent than fraudsters, but only in the academic context. We demonstrated that moral decoupling explains this effect because participants separated sexual offenders’ competence from their transgressive behavior, allowing them to conclude that although they are immoral, they are still competent. Interestingly, the moral decoupling process was used by participants only when the sexual harassment was committed in the academic context.
